# Reporting of Systematic Reviews: Better Software Required

**DOI:** 10.1371/journal.pmed.0040225

**Published:** 2007-06-26

**Authors:** Jan Brogger

This is an important paper and editorial [1,2]. Systematic reviews should be much more widespread, and not only for randomized clinical trials of clinical treatments. A paper on an elegant piece of experimental data or on epidemiological observations would be made all the more interesting if the first table were a high-quality assessment of previous studies. In fact, I would suggest that performing a systematic review should be part of a research protocol for any subject, even before the study is initiated. However, this paper confirms my suspicion that the rising popularity of “systematic reviews” has not been followed by adherence to methodological rigor.

With this background, I would like to point out one weakness that may explain part of the current quality deficit in some systematic reviews. There is a substantial lack of software that can assist in an important part of a systematic review: tracking literature searches and early phase screening. From browsing of the literature and communications with various Norwegian and Danish Cochrane collaborators (including the RevMan developers), there seems to be a limited number of tools for this use. Oftentimes, it is suggested that commercial reference management software be used, such as the popular EndNote. These types of software were not designed with systematic reviews in mind. At later stages of a review, Cochrane's RevMan is useful, but not early on.

As far as I have been able to ascertain, there are only two tools presently available. The first is EPPI-Reviewer (http://eppi.ioe.ac.uk/cms/Default.aspx?tabid=184), which is non-profit, but does not seem to be open source or available for local deployment. The second is TrialStat's SRS software (http://www.trialstat.com), which is commercial and has a substantial price tag.

I would therefore encourage researchers and institutions to contribute to the development of open-source tools for assisting in systematic reviews. I am currently writing such a simple tool, based on the open-source JabRef package (http://sourceforge.net/projects/jabref) and would welcome feedback on perceived needs and other similar projects.
